# Preparation, characterization and in vitro evaluation of phosphate-doped bioactive glass nanoparticles as promising candidates for therapeutic applications

**DOI:** 10.1186/s13065-025-01543-w

**Published:** 2025-06-19

**Authors:** Wael M. Aboulthana, Sahar M. Mousa, Gehan T. El-Bassyouni, Esmat M. A. Hamzawy, Amal G. Hussien, M. Eltohamy

**Affiliations:** 1 Biochemistry Department, Biotechnology Research Institute, National Research Centre, 33 El Bohouth St., P.O. 12622, Dokki, Cairo, Egypt; 2 Inorganic Chemistry Department, Advanced Materials Technology and Mineral Resources Research Institute, National Research Centre, 33 El Bohouth St., P.O. 12622, Dokki, Cairo, Egypt; 3 Refractories, Ceramics and Building Materials Department, Advanced Materials Technology and Mineral Resources Research Institute, National Research Centre, 33 El Bohouth St., P.O. 12622, Dokki, Cairo, Egypt; 4 Glass Research Department, Advanced Materials Technology and Mineral Resources Research Institute, National Research Centre, 33 El Bohouth St., P.O. 12622, Dokki, Cairo, Egypt

**Keywords:** Antioxidant and anti-inflammatory effects, Bioactive glass nanoparticles, Biomedical applications, In vitro bioactivity, Phosphate doping

## Abstract

This research focuses on synthesizing bioactive glass nanoparticles with different phosphate concentrations to evaluate their physicochemical characteristics and explore their biomedical potential. The nanoparticles were fabricated by melting a mixture of silica, calcium oxide, sodium oxide, and ammonium dihydrogen phosphate at 1450 °C, followed by ball milling to obtain the optimal particle size distribution. The study systematically investigated the effect of phosphate content on the nanoparticles’ structural properties and bioactive performance. Advanced characterization methods; such as XRD, FTIR, DTA, and FE-SEM–EDX were used to examine structural stability and phosphate-induced surface alterations. In vitro assays revealed dose-dependent antioxidant, anti-diabetic, anti-Alzheimer’s, anti-arthritic, and anti-inflammatory activities, with higher phosphate content enhancing bioactivity. Results demonstrated enhanced radical scavenging and enzyme inhibition in samples with higher phosphate levels. These findings suggest that phosphate-doped bioactive glass nanoparticles hold significant promise for therapeutic applications. The research advances the understanding of how synthesis parameters influence bioactivity, providing valuable insights for designing next-generation biomedical materials.

**Supplementary Information:**

The online version contains supplementary material available at 10.1186/s13065-025-01543-w.

## Introduction

Oxidative stress and excessive lipid oxidation are responsible for degenerative processes that lead to inflammation, brain damage, and protein misfolding, thereby contributing to the pathogenesis of diabetes and Alzheimer’s disorders [1, 2]. In the cholinergic synapses associated with β-amyloid plaques, acetylcholine is hydrolyzed by the enzyme acetylcholinesterase (AChE). Therefore, one therapeutic approach to managing Alzheimer’s disease is to inhibit AChE [3].

Blood glucose levels are crucially regulated by the enzymes α-amylase and α-glucosidase, which break down carbohydrates into disaccharide molecules and subsequently convert those disaccharides into monosaccharide molecules. Thus, the inhibitory action against both enzymes was evaluated to determine the anti-diabetic efficacy, which was then compared with acarbose as a reference drug [4].

Arthritis belongs to the inflammatory diseases that occur due to protein denaturation and increased activity of proteinase enzymes. Inhibition of these enzymes is considered a potential strategy for arthritis therapy [5].

Recently, the field of nanotechnology has witnessed an unprecedented surge in interest and exploration due to its potential applications in various scientific domains. Bioactive glass nanoparticles have become one of the most promising options among the many nanomaterials for a variety of biomedical uses. For therapeutic and diagnostic applications, their special qualities; such as their high surface area, adjustable composition, and bioactivity, make them especially appealing [6, 7].

Despite the growing interest in bioactive glass nanoparticles, there remains a significant gap in understanding how compositional modifications—particularly phosphate doping—affect their physicochemical properties and biological performance. While several studies have examined conventional silica-based nanoparticles, limited research has systematically investigated the influence of varying P_2_O_5_ content on the in vitro biological activities of bioactive glass systems. Furthermore, most previous works have focused on either structural analysis or cytocompatibility, with little emphasis on multifunctional therapeutic potentials such as anti-inflammatory, anti-diabetic, anti-Alzheimer’s, and antioxidant effects [8, 9]. This study aims to bridge that gap by comprehensively evaluating the relationship between phosphate incorporation and biological activity in bioactive glass nanoparticles synthesized via a top-down approach.

The current study begins a complete investigation by delving into the challenges of top-down synthesis of bioactive glass nanoparticles and their subsequent in-depth evaluation. The phrase “top-down” implies a meticulous and detailed approach, starting from the fundamental building blocks and progressing towards the final nanoparticle structure [10]. This methodology ensures a nuanced understanding of the synthesis process, allowing for precise control over the physicochemical characteristics of the nanoparticles. The main advantages of the top-down technique over the bottom-up strategy are reduced costs and greater control over the product’s shape and size [11]. The impact of different phosphorus pentoxide (P_2_O_5_) content on the properties of bioactive glass nanoparticles were studied [12]. Skerratt-Love et al. [13] argued that Na^+^ ions from the silicate sub-network compensated for the charge of P_2_O_5_. The network of charge-balancing cations is depleted when P_2_O_5_ is added to the bioactive glass nanoparticle system because some sodium ions preferentially interact with non-bridging oxygen (NBOs) in the phosphate tetrahedral.

O’Donnell et al. [14] characterized the bioactive behavior of SiO_2_-Na_2_O-CaO-P_2_O_5_ glasses during 21-day SBF testing. By varying phosphate content (0–9.25 mol.% P_2_O_5_), they observed that while initial degradation produced alkaline conditions, progressive phosphate incorporation shifted pH toward neutral due to acid–base interactions between phosphate groups and released Na^+^/Ca^2+^ ions. Significantly, compositions exceeding 3 mol. % P_2_O_5_ formed crystalline apatite within 16 h, demonstrating phosphate content’s dominant role over silicate network connectivity in controlling bioactivity within these systems*.*

Ting et al. [15] investigated the 58S sol–gel composition (60 mol.% SiO_2_, 36 mol.% CaO, 4 mol.% P_2_O_5_) and observed hydroxyapatite (HA) formation during synthesis after thermal stabilization. This preformed HA may facilitate the rapid release of calcium orthophosphate or HA nanocrystals upon exposure to SBF, rather than separate Ca^2+^ and PO_4_^3−^ ions. However, increasing the P_2_O_5_/CaO ratio suppressed HA formation. At phosphate contents > 12 mol.%, a polyphosphate glass network developed, resulting in interconnected phosphate-silica co-networks.

Adam et al. [16] found that sol–gel-derived bioglass particulates consistently demonstrated a highly amorphous structure, along with high surface area and porosity in all tested compositions. However, raising the phosphate content to 20 mol.% decreased the material’s porosity. This increase also enhanced bridging oxygen (BO) formation, strengthened the amorphous silicate network, lowered overall crystallinity, and introduced more phosphate crystallites within the glass matrix. Moreover, at this concentration, the bioglass showed reduced effectiveness in forming carbonated apatite when exposed to SBF.

Li et al. [17] examined the impact of phosphate content on the apatite-forming ability of bioactive glasses in α-MEM culture medium, as well as its role in enhancing osteogenesis both in vitro and in vivo. Their findings revealed that elevated phosphate levels not only accelerated apatite formation but also stimulated osteogenic activity. These results suggest that adjusting phosphate content in bioactive glasses can effectively modulate both apatite deposition and bone regeneration, offering a promising strategy for customized clinical applications.

Investigating the in vitro biological activities of the produced bioactive glass nanoparticles is the main goal of this work since it offers a controlled setting for evaluating the interactions between nanoparticles and biological systems at the cellular and molecular levels [18, 19]. The exploration of biological activities encompasses a range of parameters, including cytotoxicity, cellular uptake, biodegradation, and the modulation of specific cellular pathways. The Food and Drug Administration (FDA) has certified silica for its thermal resistance, inertness, low toxicity, and biocompatibility. The silanol groups (Si–OH) enable covalent interaction with other materials [20, 21]. In biological systems, the pore diameter, size, shape, and chemical modification of silica nanoparticles can affect their toxicity and bio-distribution, producing remarkable characteristics [22, 23].

The biological and therapeutic properties of silica nanoparticles include antibacterial, antitumor, anti-protozoal, and stroke treatment [24]. Their remarkable ability to enhance drug release and therapeutic procedures allows for an explanation of these biomedical activities [25]. In addition, some research has shown that silica nanoparticles have excellent biocompatibility and biosafety profiles due to their minimal or nonexistent side effects [26]. The present study not only advances the basic comprehension of bioactive glass nanoparticles but also bears noteworthy consequences for the advancement of advanced materials in the biomedical domain. The outcomes of this study may pave the way for the design and optimization of bioactive glass nanoparticles with enhanced therapeutic efficacy and reduced side effects, ultimately advancing their potential applications in medicine and healthcare. The present investigation was therefore designed to evaluate the biological activities (anti-inflammatory, anti-diabetic, anti-Alzheimer’s, anti-arthritic, and radical scavenging) of bioactive glass nanoparticles in vitro*.*

## Experimental

### Synthesis of bioactive glass nanoparticles

High-purity raw materials were used for glass synthesis: pure silica sand (SiO_2_, > 99.5%, Sigma-Aldrich) served as the source of silica; calcium oxide (CaO) was derived from analytical-grade limestone (Merck); sodium carbonate (Na_2_CO_3_, ≥ 99.9%, Sigma-Aldrich) provided Na_2_O; and ammonium dihydrogen phosphate ((NH4)H2PO4, ≥ 99.5%, Merck) supplied P_2_O_5_. The synthesis of bioactive glass began with the creation of a base glass, denoted as G0P, featuring the following composition (wt.%): SiO_2_ (47.02%), CaO (43.89%), Na_2_O (9.09%), and P_2_O_5_ (0%), serving as the reference composition. Variations of this base composition, named G1P, G2P, and G3P, were generated by introducing increasing weight percentages of P_2_O_5_ (0.90, 1.79, and 3.51%, respectively). The raw materials were meticulously mixed in appropriate proportions and underwent a conventional melting process at 1450 °C. The molar ratio between CaO and SiO_2_ was maintained at unity in all compositions, as illustrated in Table 1.

**Table 1:** The composition of prepared samples

Sample	Wt%				Mol%			
	SiO_2_	CaO	Na_2_O	P_2_O_5_	SiO_2_	CaO	Na_2_O	P_2_O_5_
G0P	47.02	43.89	9.09	0.00	45.71	45.72	8.57	0.00
G1P	46.59	43.50	9.01	0.90	45.54	45.55	8.54	0.37
G2P	46.18	43.11	8.93	1.79	45.37	45.38	8.50	0.74
G4P	45.37	42.35	8.77	3.51	45.04	45.05	8.44	1.47

Following synthesis, the resultant glass underwent ball milling in acetone, utilizing zirconia balls (Retsch GmbH, Germany) for duration of 1.5 h at 100 rpm. This step was pivotal in achieving a uniform particle size distribution and enhancing the homogeneity of the glass nanoparticles. The choice of milling parameters, encompassing the type of milling media (zirconia balls) and the duration, played an essential role in attaining the desired characteristics of the nanoparticles. This synthesis methodology ensured the production of bioactive glass nanoparticles featuring diverse P_2_O_5_ concentrations. This diversity in composition facilitated a comprehensive investigation into the influence of phosphorus content on the bioactivity and other key properties of the glass. 

### Characterization techniques

To ascertain the zeta potential and particle size distribution (PSD), a zetasizer (Nano ZS, Malvern Instruments, UK) was utilized. The phases that were obtained were recognized using the Joint Committee on Powder Diffraction Standard (JCPDS) and examined by the diffraction patterns using German XRD data with Cu-Kα radiation of 1.5406 Å (BRUKER, D8 ADVANCED Cu target). The prepared samples were examined using FTIR (Demonstrate1600, Perkin Elmer USA) at room temperature in the wavenumber range of 400–4000 cm^–1^. The thermal stability of all prepared samples were investigated by differential thermal analysis (SETARAM Labsys™), DTA where a Predetermined amount of the powder samples was fired in a platinum crucible with heating rate of 10 °C/min up to 1000 °C. These analyses provided insights into the size, morphology, crystalline structure, and chemical composition of the nanoparticles.

### Investigation of biocompatibility of prepared samples

In vitro studies are substantially less expensive and faster than *in-vivo* ones. To test biocompatibility, powdered materials were immersed in SBF for 1 week and examined for calcium phosphate development on their surfaces. To ensure accurate morphological examination, powdered sample was coated with gold/palladium by a sputtering coater (S150A-Edwards, England) with a vacuum of 0.1 Torr, 50 mA current, and 1.2 kV voltage. Coating was utilized to transform powder surfaces into conducting surfaces, which were then scanned by FE-SEM equipped with EDX to estimate the calcium phosphate ratios (Ca/P) [27, 28].

SBF is a solution with ion concentrations similar to human blood plasma that is maintained at physiological temperatures and mild pH levels. It was made using the method developed by Kokubo and Yamaguchi, which involved dissolving the reagents NaCl, NaHCO_3_, KCl, K_2_HPO_4_.3H_2_O, MgCl_2_.6H_2_O, CaCl_2_, and Na_2_SO_4_ in deionized water and using tris-(hydroxyl methyl) aminomethane and hydrochloric acid to buffer the solution to a pH of 7.4 [29]. The amount of soaking powder in the 100 mL volume of SBF remained constant, and the fluid was not refreshed during the 1-week immersion period. Samples were then removed from the SBF, thoroughly cleaned with deionized water to eliminate soluble inorganic salts and stop the reaction, and allowed to dry at room temperature. The calcium phosphate precipitation phases were assessed by analyzing the sample surface with FTIR, FE-SEM (Quanta 250 FEG, FEI, Netherlands) and EDX.

### In vitro biological assays

All biological activities were assessed using UV–VIS spectrophotometric technique (Shimadzu, UV-2401 PC) at three concentrations (250, 500, and 1000 µg/mL) of each sample based on the concept of a dose response curve, as demonstrated by El-Sayed et al*.* [30]. All assays were carried out in triplicate.

#### Antioxidant activity

By measuring the green phosphate/Mo^5+^ complex at a wavelength (λ) of 695 nm, the total antioxidant capacity (TAC) was ascertained using the method outlined by Prieto et al. [31]. Using the method proposed by Oyaizu, the iron reducing power (IRP) was determined as µg/mL [32].

#### Scavenging activity

The 1,1-Diphenyl-2-picryl-hydrazyl (DPPH) radical scavenging activity was assessed using the Rahman et al. [33] method. The scavenging activity was assayed against the 2,2′-azinobis-(3-ethylbenzothiazoline-6-sulfonic acid) (ABTS) radicals using the protocol recommended by Arnao et al. [34]. At the same concentrations as a positive control, ascorbic acid was used.

#### Anti-diabetic activity

In this study, the standard medication was Acarbose, and the inhibition percentage (%) of the α-amylase enzyme was calculated using a method developed by Wickramaratne et al. [35]. Moreover, Pistia-Brueggeman and Hollingsworth’s approach [36] was used to calculate the α-glucosidase enzyme’s inhibition percentage, using Acarbose as the standard medication.

#### Anti-Alzheimer’s activity

Using Ellman’s approach [37], the acetylcholinesterase (AChE) enzyme’s inhibition % in this investigation, using donepezil as the reference medication was assessed.

#### Anti-arthritic activity

Using diclofenac sodium as the standard non-steroidal anti-inflammatory medication, as synthesized by Meera et al*.* [38], this experiment measured the inhibition percentage (%) of protein denaturation [39] and the activity of proteinase enzyme [40].

#### Anti-inflammatory activity

The suppression of the human and ovine cyclooxygenase-1 (COX-1) and cyclooxygenase-2 (COX-2) isoenzymes, as well as the human recombinant 5-LOX enzyme, was used to assess the *in-vitro* anti-inflammatory properties. Using the COX-1 and COX-2 kit (Cayman, No.: 560131), the inhibition percentages of COX-1 and COX-2 were determined [41]. The inhibition percentages of 5-Lipoxygenase (5-LOX) were assessed using the 5-LOX kit (No. 437996, Sigma-Aldrich) as proposed by Huang et al. [42].

#### Statistical analysis

The Statistical Package for Social Sciences (SPSS for Windows, version 11.0) was used to do statistical analysis utilizing one-way analysis of variance (ANOVA) to evaluate both positive and negative correlations among in vitro biological activities. For each sample, the analysis was performed using triplicate data at the concentration (1000 µg/mL), as shown in Supplementary Table 1. To identify significant associations, a “*p*” value of less than 0.05 was chosen as the significance level.

## Result and discussion

### Characterization of prepared samples

The melting temperature was chosen using the phase diagram studied by DeCapitani and Kirschen [43], and sodium oxide (Na_2_O) was added to drop the melting temperature below 1400 °C. This strategic temperature decision, as well as the inclusion of sodium oxide, are critical to the synthesis process, assuring the best circumstances for the formation of bioactive glass. The use of sodium oxide improves glass-forming capabilities while also reducing the energy required for the melting process, thereby increasing the synthesis’s efficiency and viability.

**Fig. 1:**
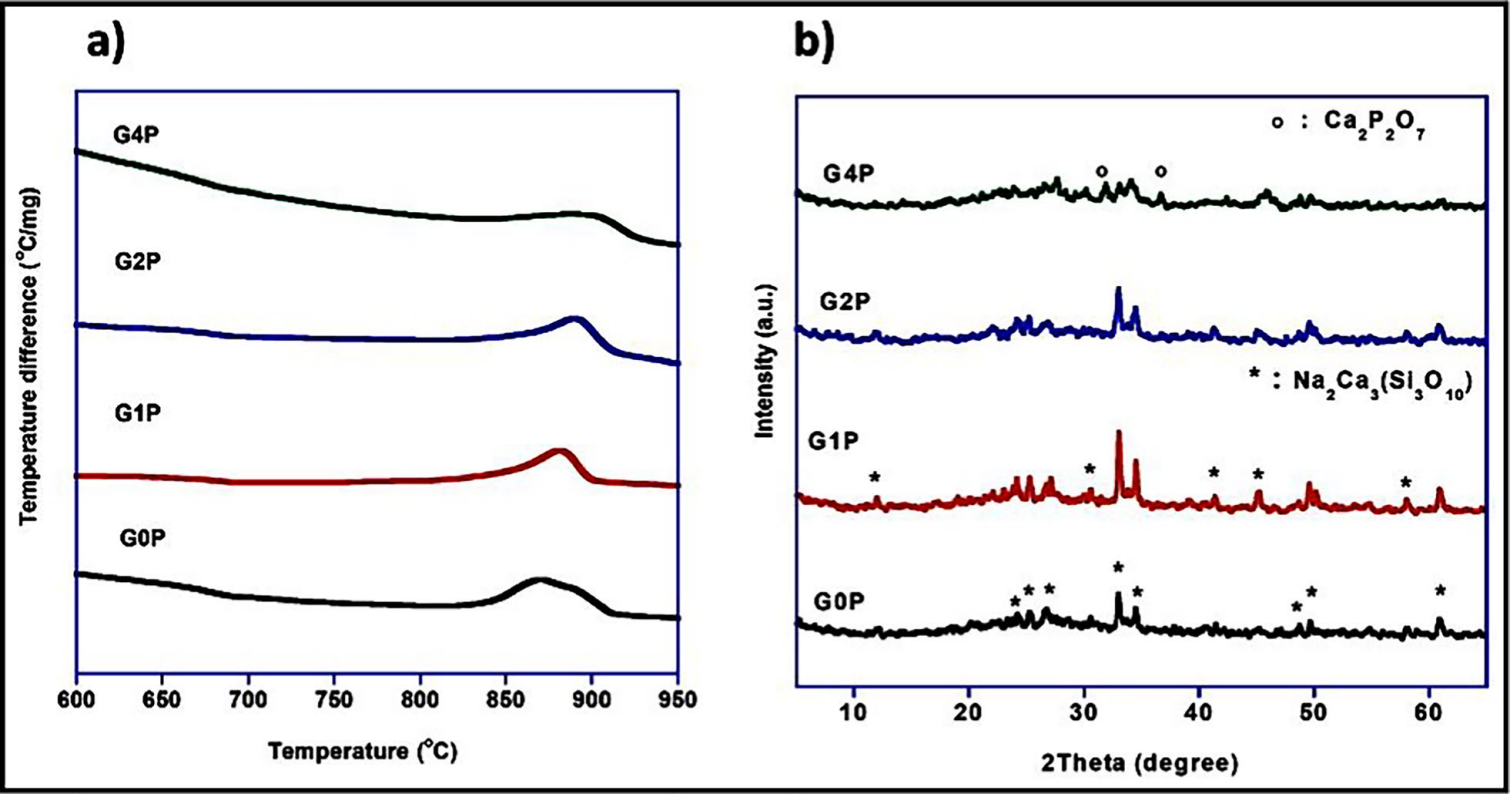
**a** DTA and **b** XRD patterns of prepared samples

Differential thermal analysis DTA was employed to analyze the thermal behavior of the bioactive glass nanoparticles during heating. The obtained data reveal crucial information about phase transitions and crystallization, providing insights into the thermal stability and processing conditions of the synthesized nanoparticles. The observed endothermic peak, which scarcely appear, corresponding to the transition from G0P to G4P compositions, was 680 and 670 °C respectively. The exothermic temperature accompanying with crystallization ranged from 865 to 895 °C, indicating a gradual increase of phosphate ratio, however, the crystallization span increase in the highest phosphate G4P-containing sample. Figure 1a showed the DTA of prepared samples. Figure 1b shows the powdered X-ray diffraction (XRD) analysis, which was performed to characterize the identification of the crystalline phases after heat-treating the glasses at 900 °C. The identified crystalline phases were Na2Ca3Si3O10 (ICCD#76-1925) and Ca_2_P_2_O_7_ (ICCD#73-0440). The presence of Na_2_Ca_3_Si_3_O_10_ is particularly noteworthy, as this phase is known to contribute to the bioactivity of the glass nanoparticles. The presence of Ca_2_P_2_O_7_ indicates the potential for phosphate ion release, which can further enhance the bioactivity of the crystalline glass nanoparticles by promoting hydroxyapatite (HA) formation and stimulating cellular responses. However, the relative abundances of these crystalline phases, as determined from the XRD data, provide insights into the composition-structure–property relationships of the crystalline bioactive glass nanoparticles. Additionally, the combination of crystalline and amorphous phases gives the nanoparticles their special qualities, allowing them to demonstrate both mechanical strength and bioactivity.

The particle size distribution (PSD) was determined to assess the size uniformity and dispersion of the bioactive glass nanoparticles. PSD data provide critical insights into the physical characteristics of the nanoparticles, influencing their biological behavior and interactions.

**Fig. 2:**
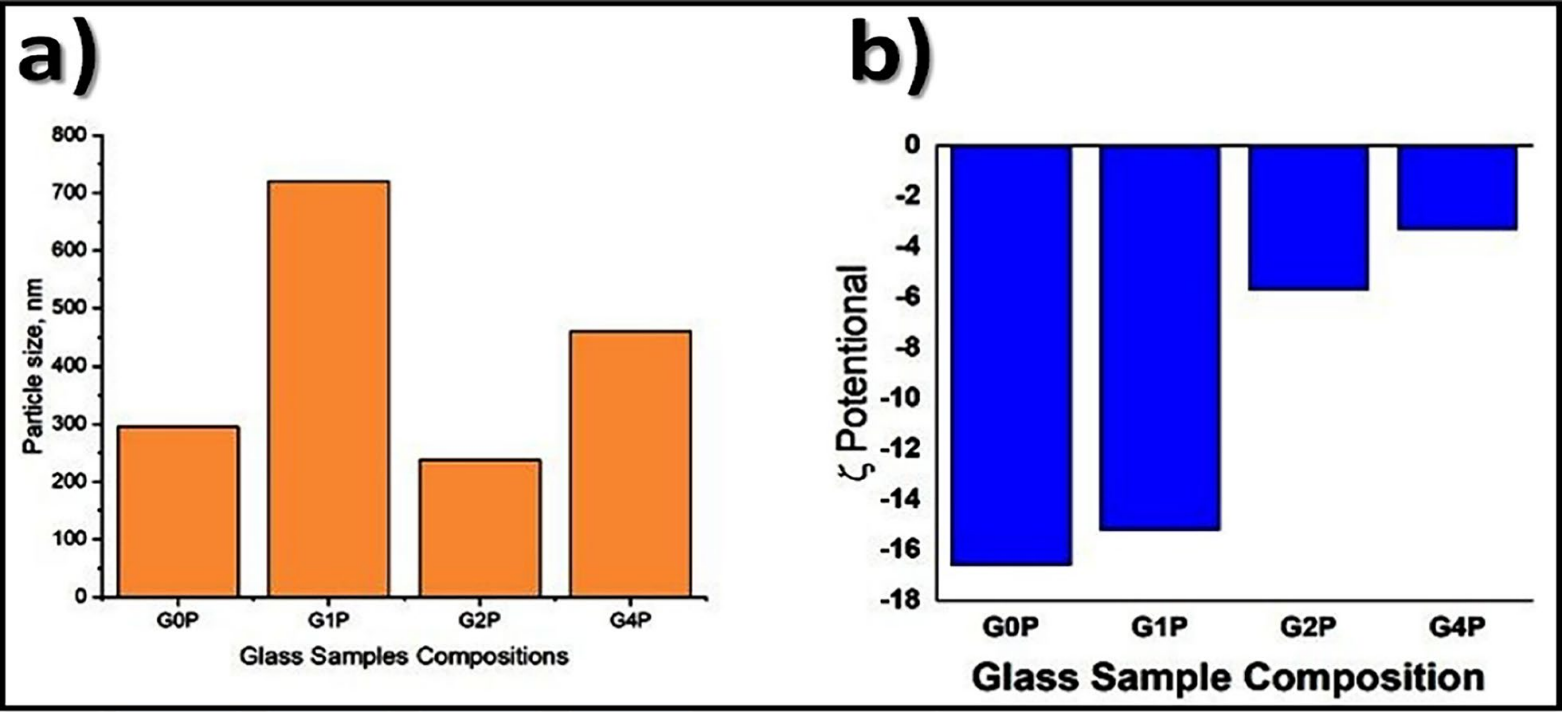
**a** Particle size distribution and** b** Zeta potential of prepared samples

Figure 2a shows the particle size distribution, and it was found that the synthesis of bioactive glass using a top-down approach yielded particles with a size distribution ranging from 300 to 700 nm. This size range is particularly significant for biomedical applications as it falls within the nano-scale regime, where materials often exhibit enhanced biological interactions and properties.

The observed particle size distribution suggests a controlled synthesis process capable of producing uniform particles within the desired range. This uniformity is crucial for ensuring consistent performance and predictable behavior of the bioactive glass in various applications, such as drug delivery systems or tissue engineering scaffolds. Furthermore, Fig. 2b, which shows zeta potential measurements of prepared samples, revealed values ranging from − 16 to − 2 with increasing phosphate content. Zeta potential is a key parameter influencing the stability and colloidal behavior of nanoparticles in solution. The negative zeta potential values indicate the presence of negatively charged surfaces on the bioactive glass particles, which can contribute to their stability by electrostatic repulsion, thereby preventing agglomeration or precipitation.

The observed trend of increasing phosphate content correlating with a decrease in the amplitude of the zeta potential suggests a surface modification effect. Phosphate ions are likely to interact with the surface of the bioactive glass particles, affecting their surface charge and, subsequently, the zeta potential. This phenomenon could have implications for the bioactivity and biocompatibility of the material, as surface properties play a crucial role in influencing cellular responses and tissue integration.

The PSD of the bioactive glass nanoparticles ranged from 300 to 700 nm, which is within the typical nano-scale range for biomedical applications. Nanoparticles in this size range are particularly relevant for a variety of reasons:

Biomedical Relevance: Nanoparticles in the range of 300–700 nm are small enough to offer enhanced surface area-to-volume ratios, which improves their biological activity and interactions with biological systems. This size range is particularly beneficial for applications in drug delivery, where nanoparticles need to efficiently interact with cell membranes or tissues, as well as in tissue engineering, where controlled degradation and biocompatibility are essential. Particles within this range can also more effectively penetrate biological barriers, such as cell membranes, promoting better therapeutic outcomes.

Controlled Synthesis: The synthesis method employed in this study, a top-down approach, was optimized to produce a uniform particle size distribution. In this process, bulk material is mechanically reduced to nanoscale dimensions. The controlled melting and ball milling techniques ensure the particles remain within the desired range of 300–700 nm. This is crucial for maintaining the desired properties of bioactive glass nanoparticles, such as their bioactivity, which can be influenced by particle size. The top-down approach is well suited for producing relatively uniform particle sizes, which are important for ensuring consistent performance in biomedical applications.

Size-Dependent Biological Interactions: The size of the nanoparticles plays a significant role in their biological interactions, including cellular uptake, biocompatibility, and bioactivity. Nanoparticles in the 300–700 nm range are large enough to avoid rapid clearance by the body’s immune system, yet small enough to interact effectively with cells. Moreover, these particles can maintain sufficient surface area for bioactive molecules to bind, enhancing their biological performance in therapeutic settings.

Zeta Potential Influence on Stability: The negative zeta potential values (− 16 to − 2 mV) indicate that the nanoparticles exhibit electrostatic repulsion, which aids in maintaining a stable dispersion in solution. The zeta potential is closely linked to particle size, with smaller nanoparticles typically showing higher zeta potential values. The relatively low negative zeta potential values observed in this study suggest that the particle surfaces possess some level of stability but are still prone to agglomeration at higher concentrations. The control of nanoparticle size in this study likely played a role in optimizing this stability, ensuring the nanoparticles can remain dispersed and effective without premature aggregation, which could compromise their biological activity.

Soaking the synthesized nanoparticles in simulated body fluid (SBF) for 1 week resulted in the formation of a hydroxyapatite (HA) crystalline phase. Hydroxyapatite, a calcium phosphate mineral similar to the mineral component of bone and teeth, is highly desirable for biomedical applications due to its excellent biocompatibility and bioactivity. The transformation of the nanoparticle into hydroxyapatite suggests its potential for promoting bone regeneration and integration when used in implantable medical devices or tissue engineering scaffolds.

**Fig. 3:**
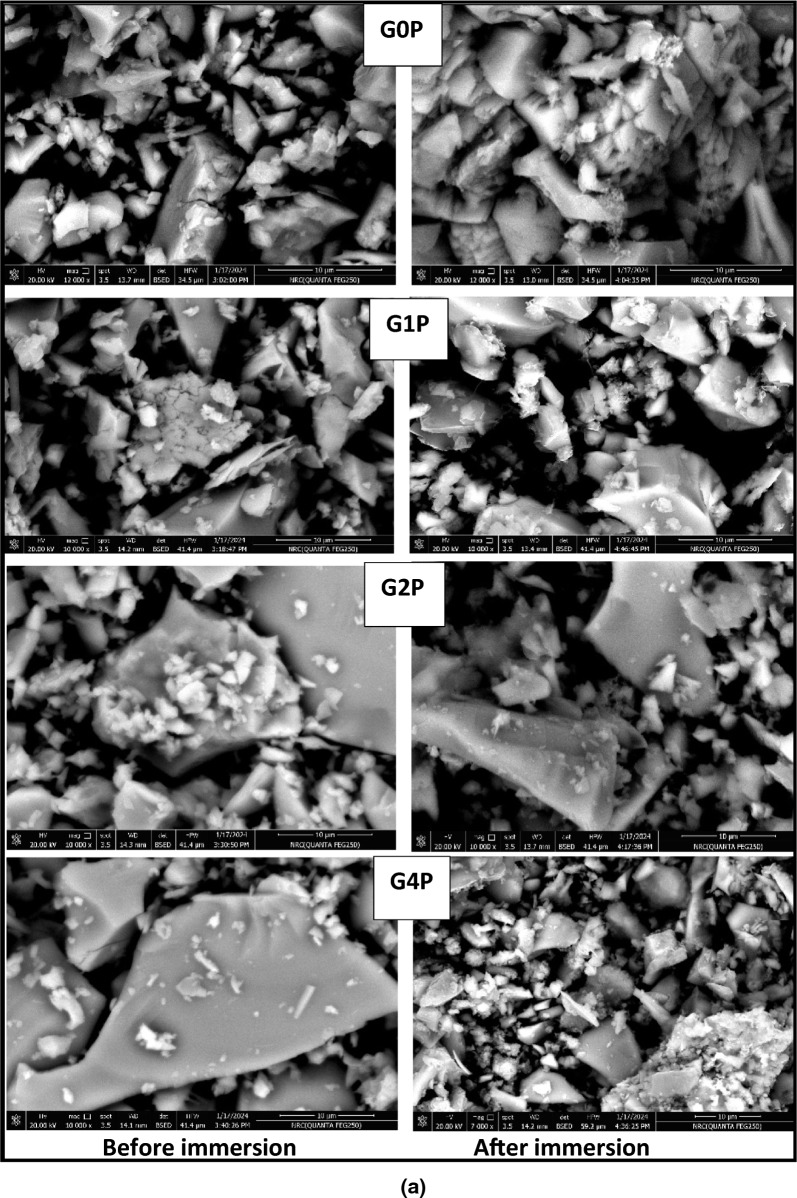

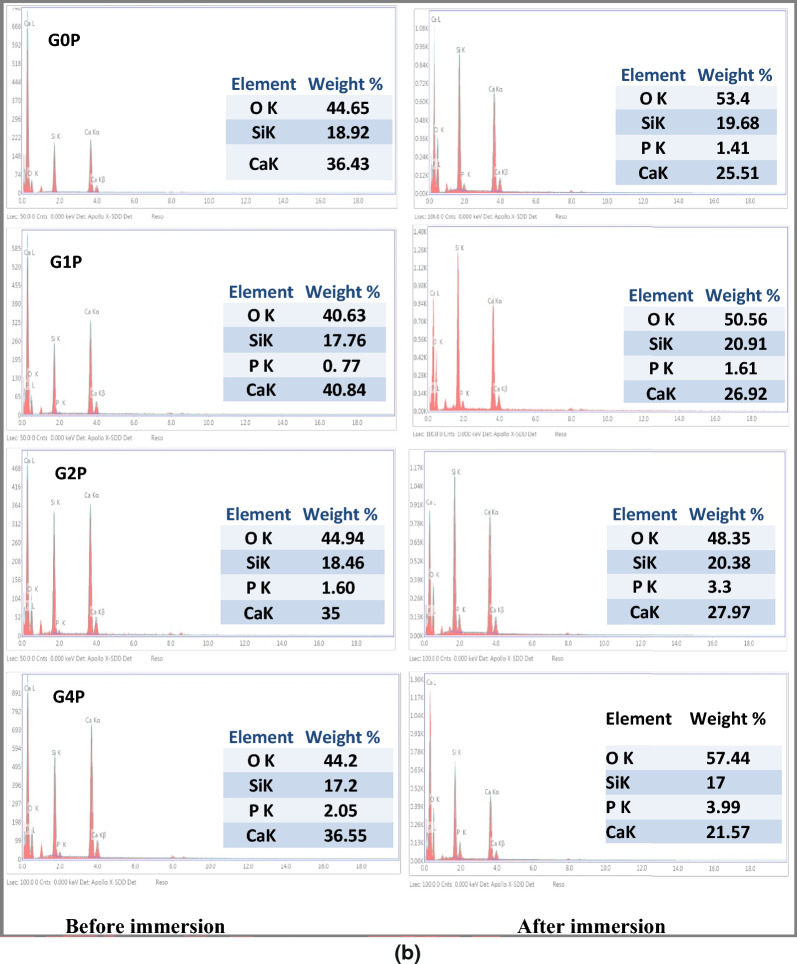
**a** SEM images of prepared samples before and after immersion in SBF. **b**. *EDX patterns before and after immersion in SBF for 1 week*

Visual proof of the morphological alterations and the development of hydroxyapatite crystals on the surfaces of the nanoparticle samples is provided by Field Emission-Scanning Electron Microscopy (FE-SEM) pictures of the samples taken both before and after soaking in SBF as illustrated in Fig. 3a. Before soaking, the nanoparticles may exhibit a uniform morphology characteristic of the synthesized material. However, after 1 week of immersion in SBF, the SEM images reveal the presence of crystalline structures resembling hydroxyapatite crystals, indicating the successful conversion of the nanoparticles into the desired phase.

Figure 3b shows the energy-dispersive X-ray spectroscopy (EDX) data, which further confirm the transformation by analyzing the elemental composition of the particles. Since calcium (Ca), phosphorous (P), and oxygen (O) are distinctive components of this mineral phase, their presence in the EDX spectra of the post-soaking samples supports the production of hydroxyapatite.

The absence of other peaks related to impurities or undesirable phases emphasizes the purity and integrity of the hydroxyapatite formed. The formation of hydroxyapatite from the nanoparticle precursor in simulated body fluid holds significant implications for biomedical applications. HA is renowned for its osteoconductive properties, which means it can promote the adhesion, proliferation, and differentiation of osteogenic cells, hence promoting bone regeneration and repair. Therefore, materials capable of inducing hydroxyapatite formation, such as the synthesized nanoparticles, may have potential applications in bone tissue engineering, orthopedic implants, and dental restoration.

Moreover, the ability to modulate the formation of HA by soaking in SBF allows for the customization of biomaterials characteristics to specific biomedical needs. By adjusting parameters such as composition, particle size, and surface characteristics, researchers can fine-tune the kinetics and extent of hydroxyapatite formation, optimizing the material’s performance in various biological environments.

### In vitro biological activities

#### Antioxidant activity

Common conditions including rheumatoid arthritis, atherosclerosis, and cancer are believed to be caused by excessive lipid oxidation and inflammation. Reducing these oxidation processes with exogenous antioxidant consumption may be the primary approach to treating and preventing these diseases [44–47]. TAC and IRP were measured to evaluate the antioxidant activity of bioactive glass nanoparticles at three concentrations (250, 500, and 1000 µg/mL). According to the findings in Table 2, there was a concentration-dependent connection between TAC and IRP, with both increasing as sample concentration rose. In particular, samples at 1000 µg/mL showed the strongest antioxidant activity when compared to those at lower doses (250 and 500 µg/mL). This outcome is consistent with Ferraris et al. [48], who noted that the presence of a high hydroxylation degree is a key factor in the antioxidant ability of bioactive glasses. In comparison to the other samples, sample G4P demonstrated the highest antioxidant activity. At the highest concentration studied (1000 µg/mL), this sample exhibited a higher TAC (77.03 ± 0.08 mg gallic acid/g) and IRP (65.94 ± 0.13 µg/mL). In contrast, at the same concentration, the TAC and IRP of standard ascorbic acid were 96.29 ± 0.10 mg gallic acid/g and 82.42 ± 0.16 µg/mL, respectively. The antioxidant activity is entirely dependent on the concentrations of the ingredients in each sample. The samples with weight percentages of P_2_O_5_ showed superior antioxidant activity compared to free P_2_O_5_ at all concentrations tested. This could be attributed to the increased surface density of the active molecules [49]. No matter where they come from, using large amounts of natural antioxidants can be hazardous. In this context, it has been noted that high phenolic intakes may pose health risks because of their interactions with proteins. Polyphenolic compounds have the ability to disable enzymes, for instance. Consequently, biomaterial-assisted methods must be used to focus the delivery of antioxidants to certain sites without any restrictions [50].

**Table 2:** Antioxidant and scavenging activities of various concentrations of bioactive glass nanoparticles

Sample	Conc. (µg/mL)	Antioxidant activity		Scavenging activity	
		TAC (mg gallic acid/g)	IRP (µg/mL)	DPPH	ABTS
				Inhibition (%)	
G0P	250	17.24 ± 0.02	14.76 ± 0.03	12.24 ± 0.02	19.56 ± 0.02
	500	30.17 ± 0.03	25.82 ± 0.05	21.42 ± 0.03	34.22 ± 0.04
	1000	52.80 ± 0.05	45.19 ± 0.09	37.49 ± 0.05	59.89 ± 0.07
G1P	250	19.83 ± 0.02	16.97 ± 0.03	14.08 ± 0.02	22.49 ± 0.03
	500	34.70 ± 0.03	29.70 ± 0.06	24.63 ± 0.03	39.36 ± 0.05
	1000	60.72 ± 0.06	51.97 ± 0.10	43.11 ± 0.06	68.88 ± 0.08
G2P	250	20.12 ± 0.02	17.22 ± 0.03	14.29 ± 0.02	22.83 ± 0.03
	500	35.22 ± 0.04	30.14 ± 0.06	25.00 ± 0.04	39.95 ± 0.05
	1000	61.63 ± 0.06	52.75 ± 0.10	43.75 ± 0.06	69.91 ± 0.08
G4P	250	25.15 ± 0.03	21.53 ± 0.04	17.86 ± 0.03	28.53 ± 0.03
	500	44.02 ± 0.04	37.68 ± 0.07	31.25 ± 0.04	49.94 ± 0.06
	1000	77.03 ± 0.08	65.94 ± 0.13	54.69 ± 0.08	87.39 ± 0.10
Ascorbic Acid	250	31.44 ± 0.03	26.91 ± 0.05	25.85 ± 0.05	30.17 ± 0.01
	500	55.02 ± 0.06	47.10 ± 0.09	45.23 ± 0.09	52.80 ± 0.02
	1000	96.29 ± 0.10	82.42 ± 0.16	79.16 ± 0.15	92.40 ± 0.04

Assays were run in threefold (*n* = 3), and the results were calculated from three replicates and presented as mean ± standard error (SE), Orange cell indicates the most active sample

#### Scavenging activity

Antioxidants have been suggested to play a crucial role in protecting cells from damage by scavenging harmful free radicals. Continuous consumption of exogenous antioxidants has been linked to a reduced risk of developing many chronic diseases [51]. The scavenging activity of bioactive glass nanoparticles was evaluated by testing the inhibitory effect of the samples against DPPH and ABTS radicals at three different concentrations (250, 500, and 1000 µg/mL). Table 2 demonstrates that the scavenging activity against both DPPH and ABTS radicals increased in all studied samples as their concentrations increased. At a concentration of 1000 µg/mL, each sample exhibited the highest scavenging activity compared to the other concentrations (250 and 500 µg/mL). This could be attributed to the interaction of specific moieties on the surfaces of the tested bioactive glasses, leading to the abstraction of the hydrogen atom of –OH and the production of a siloxyl radical (Si–O^•^) and water, or the trapping of HO^•^ by Si with the formation of a pentacoordinate silicon complex [52]. Additionally, the silanol-OH groups play a role in scavenging, as evidenced by the noticeable decrease in activity after extensive and largely irreversible dehydroxylation of the silica surface [53–55]. The G4P sample demonstrated the highest scavenging activity compared to the other samples, exhibiting the highest inhibitory effect against DPPH (54.69 ± 0.08%) and ABTS radicals (87.39 ± 0.10%) at the highest concentration (1000 µg/mL) compared to the other samples and concentrations. This may be due to the presence of active ingredients in the hydroxyapatite that can donate hydrogen to a free radical in order to remove the electron responsible for the radical’s reactivity [56, 57]. All samples showed concentration-dependent chelation activities, with the G4P sample showing stronger activity as an antioxidant [58]. At the same concentration, the standard ascorbic acid inhibited the DPPH and ABTS radicals by 79.16 ± 0.15 and 92.40 ± 0.04%, respectively.

#### Anti-diabetic activity

A chronic metabolic disorder marked by high glucose levels is diabetes mellitus (DM) [59]. Blood glucose levels are mostly controlled by the enzymes α-amylase and α-glucosidase, which break down carbs into disaccharides and turn those disaccharides into monosaccharides, respectively. To treat hyperglycemia, one therapeutic approach is to inhibit these enzymes [60]. Throughout the current experiment, it was found that none of the samples significantly inhibited the activity of the enzymes α-amylase and α-glucosidase at any of the quantities that were examined (250, 500, and 1000 µg/mL) (Table 3). In contrast, at the same concentrations, the standard Acarbose showed inhibited activity of α-amylase by 25.82 ± 0.02, 48.11 ± 0.20, and 89.00 ± 0.36, respectively and inhibited activity of α-glucosidase by 11.64 ± 0.02, 21.68 ± 0.09, and 40.11 ± 0.16, respectively.

**Table 3:** The in vitro anti-diabetic and anti-alzheimer activities of various concentrations of bioactive glass nanoparticles

Sample	Conc. (µg/mL)	Anti-diabetic activity		Anti-Alzheimer activity
		α-amylase	α-glucosidase	AChE
		Inhibition (%)		
G0P	250	27.18 ± 0.02	12.25 ± 0.02	22.25 ± 0.03
	500	27.59 ± 0.02	12.43 ± 0.20	38.94 ± 0.06
	1000	27.68 ± 0.02	12.48 ± 0.02	68.14 ± 0.11
G1P	250	27.59 ± 0.02	12.43 ± 0.02	16.75 ± 0.03
	500	28.07 ± 0.02	12.65 ± 0.02	33.44 ± 0.06
	1000	28.10 ± 0.02	12.66 ± 0.02	62.64 ± 0.11
G2P	250	27.65 ± 0.02	12.46 ± 0.02	12.25 ± 0.03
	500	27.70 ± 0.02	12.49 ± 0.02	28.94 ± 0.06
	1000	27.71 ± 0.02	12.49 ± 0.02	58.14 ± 0.11
G4P	250	27.72 ± 0.02	12.49 ± 0.02	17.75 ± 0.03
	500	27.77 ± 0.02	12.52 ± 0.02	34.44 ± 0.06
	1000	27.96 ± 0.02	12.60 ± 0.02	63.64 ± 0.11
STD	Conc. (µg/mL)	Acarbose		Donepezil
	250	25.82 ± 0.02	11.64 ± 0.02	26.75 ± 0.03
	500	48.11 ± 0.20	21.68 ± 0.09	43.44 ± 0.06
	1000	89.00 ± 0.36	40.11 ± 0.16	72.64 ± 0.11

Assays were run in threefold (*n* = 3), and the results were calculated from three replicates and presented as mean ± standard error (SE), Orange cell indicates the most active sample

#### Anti-Alzheimer’s activity

Alzheimer’s disease causes neurodegeneration, which leads to death and cognitive deterioration. One of the main causes of Alzheimer’s disease is the activation of the AChE enzyme. Inhibiting this enzyme is therefore a useful therapeutic approach for the management of the illness [61]. The data in Table 3 demonstrated that all substances’ inhibitory effects on the AChE enzyme grew as concentrations rose. Among the samples, the one that had the strongest inhibitory impact on this enzyme was G0P. Therefore, this sample inhibited the activity of the AChE enzyme by 68.14 ± 0.11% at the highest concentration (1000 µg/mL) compared to other concentrations (250 and 500 µg/mL). This might be primarily related to role of the nanoparticles in increasing the affinity for AChE adsorption. Therefore, the AChE may have the potential to be used as a biomarker for nanoparticles [62]. In contrast, at the same studied concentrations (250, 500 and 1000 µg/mL), the standard donepezil showed inhibitory effects of 26.75 ± 0.03, 43.44 ± 0.06, and 72.64 ± 0.11%, respectively.

**Fig. 4:**
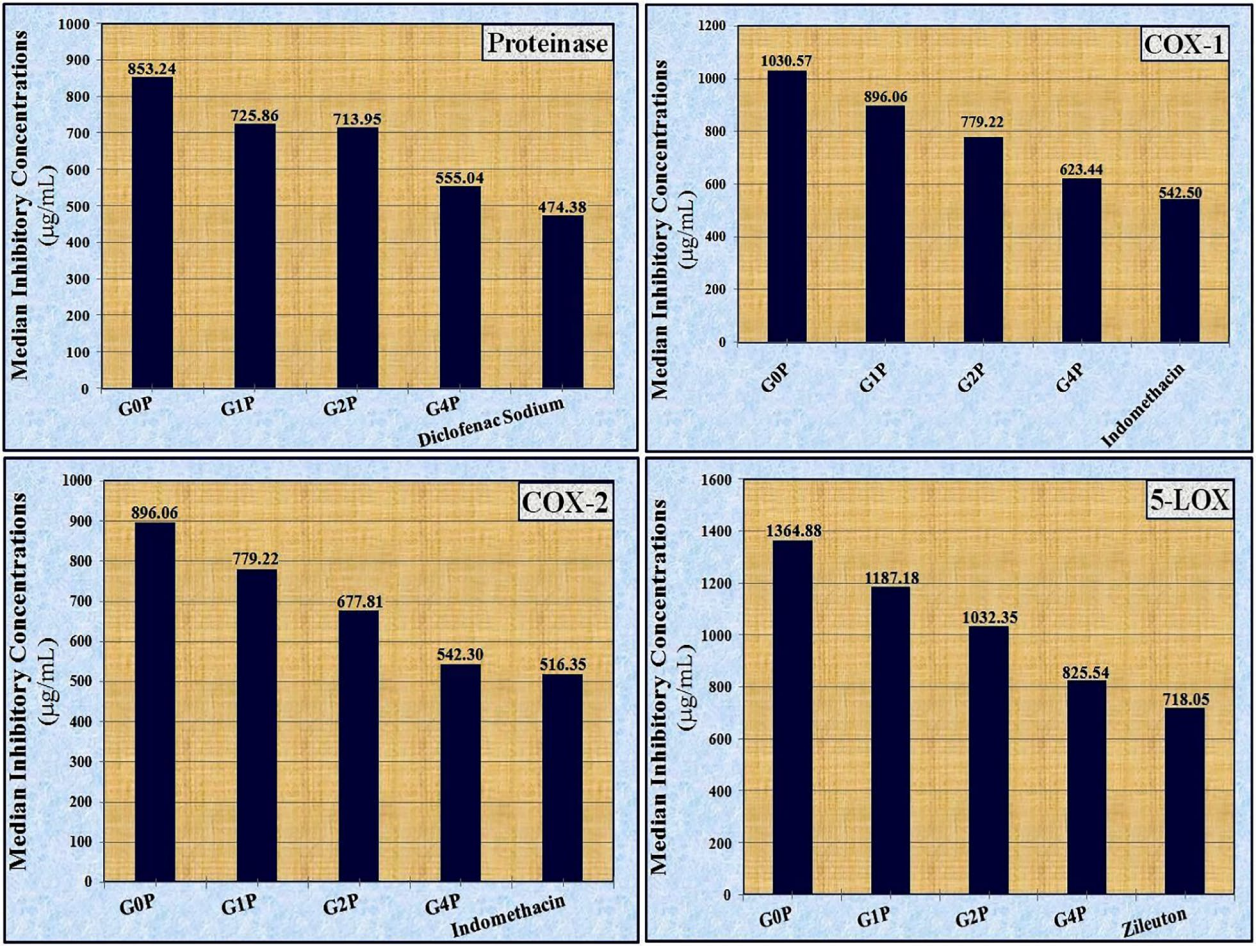
The median inhibitory concentrations (IC_50_) of bioactive glass nanoparticles against AChE, proteinase and markers of the inflammatory reactions (COX-1, COX-2 and 5-LPO) compared to their standards

Figure 4 shows the IC_50_ of bioactive glass nanoparticles against AChE in comparison to the donepezil standard. Data compiled in Table 4 showed that a low IC_50_ indicates higher anti-Alzheimer’s activity. The lowest IC_50_ value was detected with the sample G0P (561.80 µg/mL) followed by the sample G4P (704.23 µg/mL), and then the sample G1P (746.27 µg/mL) compared to the standard donepezil (467.29 µg/mL) which showed the lowest IC_50_ value.

**Table 4:** The median inhibitory concentrations (IC_50_) of bioactive glass nanoparticles against AChE, proteinase and markers of the inflammatory reactions (COX-1, COX-2 and 5-LPO)

Sample	Anti-Alzheimer activity	Anti-arthritic activity	Anti-inflammatory activity		
	AChE	Proteinase	COX-1	COX-2	5-LOX
	IC_50_ (µg/mL)				
G0P	561.80	853.24	1030.57	896.06	1364.88
G1P	746.27	725.87	896.06	779.22	1187.18
G2P	1020.41	713.95	779.22	677.81	1032.35
G4P	704.23	555.04	623.44	542.30	825.54
STD	Donepezil	Diclofenac Sodium	Indomethacin		Zileuton
	467.29	474.38	542.50	516.35	718.05

Orange cell indicates the most active sample

#### Anti-arthritic activity

One of the key signs of arthritis is inflammation, which itself defines the condition [63]. The apparent potential for anti-inflammatory activity is referred to by the analyzed materials’ capacity to suppress proteinase denaturation and proteinase enzymes, which are important characteristics and indicators for the occurrence of inflammatory disorders, including arthritis [64]. The alteration of forces including disulfide bridges, ionic contacts, electrostatic forces, and hydrogen bonds that stabilize proteins and are necessary for their structure and function is a significant part of protein denaturation. Moreover, anti-inflammatory medications exhibit dose-dependent inhibition of protein denaturation [65]. Regarding the data on anti-arthritic activity obtained during the current study, the data in Table 4 showed that the inhibitory effect of all samples on protein denaturation and the activity of the proteinase enzyme increased with increasing concentrations. This finding is consistent with the postulation by Lobel and Hench [66] that bioactive glasses can act as “friendly hosts” for adsorbed proteins. Additionally, the presence of calcium ions in the structure may lead to different cross-linking densities and bond strengths between the cross-linked protein chains, which can also affect enzymatic degradation [67]. The sample G4P showed the highest inhibitory effect on these measurements compared to the other samples. This sample, at a concentration of 1000 µg/mL, inhibited protein denaturation by 76.62 ± 0.06% and inhibited the activity of the proteinase enzyme by 74.12 ± 0.06% compared to other concentrations (250 and 500 µg/mL). This might be related to the potential created by hydroxyapatite to improve the cross-link density and the physical crosslinking, thus improving the mechanical strength. This improvement could be attributed to an increase in its specific surface energy, which increased the thickness of the porous wall and reduced the brittleness of the hydroxyapatite [68]. Moreover, the Ca^2+^ released from hydroxyapatite increased the degree of cross-linking and the formation of hydrogen bonds [69]. In contrast, at the same studied concentrations (250, 500 and 1000 µg/mL), the standard Diclofenac Sodium inhibited protein denaturation by 28.85 ± 0.05, 50.48 ± 0.09, and 88.34 ± 0.15%, respectively and inhibited proteinase enzyme by 26.35 ± 0.05, 47.98 ± 0.09, and 85.84 ± 0.15%, respectively. Figure 4 shows the IC_50_ values of bioactive glass nanoparticles against the activity of the proteinase enzyme. As compiled in Table 4, the lowest IC_50_ value was found with the sample G4P (555.04 µg/mL) followed by the sample G2P (713.95 µg/mL) and then the sample G1P (725.87 µg/mL) compared to the standard Diclofenac Sodium (474.38 µg/mL) which showed the lowest IC_50_ value.

#### Anti-inflammatory activity

An enzyme called cyclooxygenase (COX) facilitates the transformation of arachidonic acid (AA) into prostaglandins (PGs). The two isoforms of COX, COX-1 and COX-2, are very different from one another [70]. The production of arachidonic acid depends on the cyclooxygenases (COX-1 and COX-2) and lipoxygenase (5-LOX), which are well-known pro-inflammatory enzymes [71]. As a result, anti-inflammatory drugs are regularly screened and evaluated using these enzymes. Furthermore, 5-LOX stimulates the release of eicosanoids via the second major metabolic pathway. The end product of the 5-LOX pathway, leukotriene B4, mediates a number of inflammatory conditions and sensitive illnesses. Lowering leukotriene levels through 5-LOX inhibition could help minimize the risk of cardiovascular and gastrointestinal problems [72]. Due to the various side effects and limited activities of synthetic drugs, the search for natural anti-inflammatory agents is essential as they could be safer and more effective than synthetic medicines [73].

Regarding the data on anti-inflammatory activity obtained during the present study, the data in Table 5 showed that the inhibitory effect of all the studied samples on the activities of the COX-1, COX-2, and 5-LOX enzymes increased with increasing concentrations. This was in accordance with Agarwal et al. [74], who demonstrated that bioactive glasses exhibit anti-inflammatory activity due to the presence of nanoparticles, which have a large surface area to volume ratio. Therefore, they are better at blocking inflammation-enhancing enzymes compared to their bulk counterparts. The sample G4P showed the highest inhibitory effect on the activities of these enzymes compared to the other samples. This sample at the highest concentration (1000 µg/mL) inhibited the activities of COX-1, COX-2 and 5-LOX by 76.23 ± 0.08, 87.66 ± 0.09 and 57.58 ± 0.13%, respectively compared to other concentrations (250 and 500 µg/mL) and the other studied samples. In contrast, at the same concentration (1000 µg/mL), the standard indomethacin inhibited activities of the COX-1 and COX-2 by 87.66 ± 0.09 and 87.39 ± 0.09%, respectively. Regarding the activity of the 5-LOX enzyme, the standard Zileuton inhibited the activity of this enzyme by 66.22 ± 0.15%.

**Table 5:** The in vitro anti-arthritic and anti-inflammatory activities of various concentrations of bioactive glass nanoparticles

Sample	Conc. (µg/mL)	Anti-arthritic activity		Anti-inflammatory activity		
		Protein Denaturation	Proteinase	COX-1	COX-2	5-LOX
		Inhibition (%)				
G0P	250	17.15 ± 0.01	14.65 ± 0.01	12.13 ± 0.01	13.95 ± 0.01	9.16 ± 0.02
	500	30.01 ± 0.03	27.51 ± 0.03	22.43 ± 0.02	25.80 ± 0.03	16.95 ± 0.04
	1000	52.51 ± 0.04	50.01 ± 0.04	41.50 ± 0.04	47.73 ± 0.05	31.35 ± 0.07
G1P	250	19.72 ± 0.02	17.22 ± 0.02	13.95 ± 0.01	16.04 ± 0.02	10.53 ± 0.02
	500	34.51 ± 0.03	32.01 ± 0.03	24.40 ± 0.02	28.07 ± 0.03	18.43 ± 0.04
	1000	60.39 ± 0.05	57.89 ± 0.05	42.71 ± 0.04	49.11 ± 0.05	32.26 ± 0.07
G2P	250	20.01 ± 0.02	17.51 ± 0.02	16.04 ± 0.02	18.44 ± 0.02	12.11 ± 0.03
	500	35.03 ± 0.03	32.53 ± 0.03	28.07 ± 0.03	32.28 ± 0.03	21.20 ± 0.05
	1000	61.29 ± 0.05	58.79 ± 0.05	49.11 ± 0.05	56.48 ± 0.06	37.10 ± 0.08
G4P	250	25.02 ± 0.02	22.52 ± 0.02	20.05 ± 0.02	23.05 ± 0.02	15.14 ± 0.03
	500	43.78 ± 0.04	41.28 ± 0.04	39.09 ± 0.04	44.96 ± 0.04	29.53 ± 0.07
	1000	76.62 ± 0.06	74.12 ± 0.06	76.23 ± 0.08	87.66 ± 0.09	57.58 ± 0.13
STD	Conc. (µg/mL)	Diclofenac Sodium		Indomethacin		Zileuton
	250	28.85 ± 0.05	26.35 ± 0.05	23.05 ± 0.02	24.21 ± 0.02	17.41 ± 0.04
	500	50.48 ± 0.09	47.98 ± 0.09	44.96 ± 0.04	45.99 ± 0.05	33.96 ± 0.08
	1000	88.34 ± 0.15	85.84 ± 0.15	87.66 ± 0.09	87.39 ± 0.09	66.22 ± 0.15

Assays were run in threefold (*n* = 3), and the results were calculated from three replicates and presented as mean ± standard error (SE), Orange cell indicates the most active sample

Figure 4 shows the IC_50_ values against markers of the inflammatory reactions (COX-1, COX-2 and 5-LPO). As depicted in Table 4, the lowest IC_50_ values against COX-1, COX-2 and 5-LOX enzymes were found with the sample G4P (623.44, 542.30 and 825.54 µg/mL, respectively) followed by the sample G2P (779.22, 677.81 and 1032.35 µg/mL, respectively) and then the sample G1P (896.06, 779.22 and 1187.18 µg/mL, respectively) compared to the corresponding standard (542.50, 516.35 and 718.05 µg/mL, respectively) which showed a lower IC_50_ value.

Table 6 indicates that at equal concentrations (1000 µg/mL) of the investigated samples, there is a positive correlation between the measurements of the antioxidant (TAC and IRP), scavenging (DPPH and ABTS), anti-arthritic (inhibition % of protein denaturation and activity of proteinase activity), and anti-inflammatory (inhibition % of COX-1, COX-2, and 5-LOX enzymes) activities. This correlation is significant at the *p* ≤ level. It was observed that as antioxidant and scavenging activities increase, so do the anti-arthritic and anti-inflammatory activities. This confirms the results showing a strong correlation between increasing antioxidant and scavenging effectiveness and the inhibitory actions against these enzymes. However, there was no significant link between the inhibitory impact and other in vitro biological activities when measured against the anti-diabetic (α-amylase and α-glucosidase) and anti-Alzheimer’s activities (AChE).

**Table 6:**
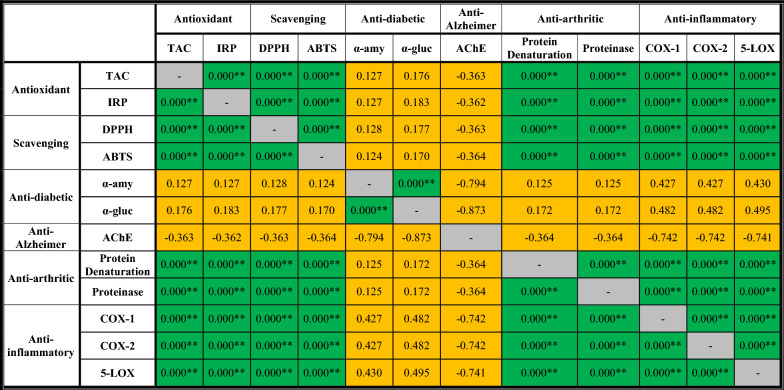
The statistical correlations among the different in vitro biological activities of bioactive glass nanoparticles at equal concentrations (1000 µg/mL)

Orange cell indicates a non-significant correlation (*p* ≤ 0.05), and green cell indicates a positive correlation (*p* ≤ 0.01)

## Conclusion

In this study, we conducted an extensive investigation into top-down synthesis techniques and evaluated the biological activities of bioactive glass nanoparticles, with a focus on the in vitro performance. Through meticulous experimentation, we gained valuable insights into the synthesis process, physicochemical properties, and biological behavior of these promising nanomaterials. Our results demonstrated that top-down synthesis provides precise control over particle size distribution and surface characteristics, yielding nanoparticles with favorable traits for biomedical applications. The synthesized bioactive glass nanoparticles exhibited a particle size distribution between 300 and 700 nm, which is ideal for enhancing cellular interactions and tissue integration. Additionally, we examined the effect of phosphate content on the zeta potential of the nanoparticles, emphasizing the significance of surface modification in tailoring biological responses. The observed variations in zeta potential offer useful information for optimizing the design of bioactive glass nanoparticles to achieve targeted biomedical outcomes.

In vitro biological testing confirmed the bioactivity of the synthesized nanoparticles, evidenced by the formation of hydroxyapatite crystals upon immersion in simulated body fluid. This transformation highlights the potential of bioactive glass nanoparticles to promote antioxidant, scavenging, anti-arthritic, and anti-inflammatory activities.

Overall, our study contributes to the growing knowledge of bioactive glass nanoparticles and their biomedical applications. By detailing the synthesis process, characterizing their physicochemical properties, and exploring the *in-vitro* biological activities, we have laid the foundation for future research aimed at unlocking the full potential of these innovative nanomaterials to improve human health and advance medical treatments. Limitations regarding in vitro applicability, particle agglomeration, and lack of long-term cytotoxicity testing have been added to the Conclusion section.

### Recommendation

Further in vivo studies are recommended to be carried out to simulate the complexities of biological environments within living organisms, especially rats. These studies can offer insights into the nanoparticles’ behavior, biocompatibility, and potential therapeutic effects in a more holistic context.

## Supplementary Information

Additional file 1.

## Data Availability

Data is provided within the manuscript as a supplementary information file.
