# Supplementary material for: Preparation, characterization and in vitro evaluation of phosphate-doped bioactive glass nanoparticles as promising candidates for therapeutic applications
Source: BMC Chem. 2025 Jun 19;19(1):170. doi: 10.1186/s13065-025-01543-w (PMC12180270; doi:10.1186/s13065-025-01543-w)
Supplement: Supplementary file 1 — Additional file 1. [file 13065_2025_1543_MOESM1_ESM.doc]

**Supplementary Material**

**Detailed Methods**

***In vitro* biological Assays**

All biological activities were assessed at three concentrations (250, 500, and 1000 µg/mL) in triplicate for each sample based.

**1. Antioxidant activity**

By measuring the green phosphate/Mo^5+^ complex at a wavelength (λ) of 695 nm, the total antioxidant capacity (TAC) was ascertained using the method outlined by **Prieto *et al*.** **[31]**. The samples were combined with a reagent solution of 4 mM ammonium molybdate, 28 mM sodium phosphate, and 0.3 N sulfuric acid at each concentration. The blank sample was substituted with 80% methanol. After sealing, the tubes were placed in a boiling water bath and left for ninety minutes. Absorbance was measured at 695 nm against the blank after cooling to ambient temperature. At the same amounts as the standard, ascorbic acid was used. In milligrams of gallic acid equivalent per gram of weight, the outcome was reported.

Using the method proposed by Oyaizu, the iron reducing power (IRP) was determined as µg/mL **[32]**. In brief, 1mL of the tested sample (at each concentration) was combined with 1mL of 200mM sodium phosphate buffer (pH 6.6) and 1mL of 1% potassium ferricyanide (C_6_N_6_FeK_3_). The mixture was then incubated at 50°C for 20 minutes, followed by the addition of 1mL of 10% trichloroacetic acid (C_2_HCl_3_O_2_). After centrifugation at 2000rpm for 10 minutes, the upper layer solution (2.5 mL) was mixed with 2.5 mL of double deionized water and 1mL of fresh 0.1% ferric chloride (FeCl_3_). The absorbance was measured at 700 nm against a blank prepared without the sample. Ascorbic acid (C_6_H_8_O_6_) was used at the same concentrations as a standard. A high absorbance at 700 nm designates a higher reducing power in the reaction mixture.

**2. Scavenging activity**

*- DPPH radical-scavenging activity*

The 1,1-Diphenyl-2-picryl-hydrazyl (DPPH) radical scavenging activities were assessed using the **Rahman *et al*.** **[33]** method, which involves mixing two milliliters of a DPPH solution (100 µM) in ethanol with two milliliters of the sample (at each concentration). An antioxidant that can donate a hydrogen atom to a solution containing DPPH- can reduce the stable free radical, causing the solution to change color from violet to pale yellow. Each concentration's reaction mixture was vortexed vigorously before being allowed to sit at room temperature for half an hour in the dark. After that, the absorbance was spectrophotometrically measured at 518 nm in relation to an ethanol blank. The test was conducted concurrently with the addition of 2 mL of ethanol as the control. At the same concentrations as a positive control, ascorbic acid was employed. It was determined what percentage of the DPPH free radical was inhibited.

- *ABTS radical scavenging assay*

The protocol recommended by **Arnao *et al*.** **[34]** was followed for the 2,2'-azinobis-(3-ethylbenzothiazoline-6-sulfonic acid) (ABTS) test. ABTS solution (7 mM) and potassium persulfate (K_2_S_2_O_8_) solution (2.4 mM) were examples of stock solutions. The two stock solutions were combined in equal amounts to create the working solution, which was then left to react for 14 hours at room temperature in a dark environment. Then, using a spectrophotometer, the solution was diluted by combining 1 mL of ABTS solution with 60 mL of methanol to achieve an absorbance of 0.706 ± 0.01 units at 734 nm. For every trial, a new ABTS solution was created. After allowing the tested samples (at each concentration) to react with 1 mL of the ABTS solution for 7 minutes, the absorbance was measured with a spectrophotometer at 734 nm. The samples' ability to scavenge ABTS was contrasted with that of ascorbic acid (at the same doses).

**3. Anti-diabetic activity**

In this study, the standard medication was Acarbose, and the inhibition percentage (%) of the α-amylase enzyme was calculated using a method developed by **Wickramaratne *et al*.** **[35]**. 0.5 mL of each sample (at each concentration) was mixed with 0.5 mL of α-amylase solution (0.5 mg/mL) and buffer [Na_2_HPO_4_/NaH_2_PO_4_ (0.02 M), NaCl (0.006 M) at pH 6.9] during the experiment. 200 μL of starch solution (1% in water (w/v) buffer [Na_2_HPO_4_/NaH_2_PO_4_ (0.02 M), NaCl (0.006 M) at pH 6.9) was then added to the mixture after it had been at room temperature for ten minutes. By adding 200 μL of DNSA (coloring) reagent (12 g of sodium potassium tartrate tetrahydrate in 8.0 mL of 2 M NaOH and 20 mL of 96 mM DNSA solution), the reaction was stopped. After ten minutes in a boiling water bath (100°C), the mixture was allowed to cool to room temperature and diluted with five milliliters of distilled water in the test tubes. The absorbance was determined with a UV-Visible spectrophotometer at 540 nm.

**Pistia-Brueggeman and Hollingsworth's** approach **[36]** was used to calculate the α-glucosidase enzyme's inhibition percentage, using Acarbose as the standard medication. Ten microliters of each sample (at each concentration) were pre-mixed with five microliters of the α-glucosidase solution (10 units/mL, 0.1mol/L potassium phosphate buffer, pH 6.8). To initiate the reaction, 10 μL of p-nitro phenyl glucopyranoside (*p*NPG, 10 mmol/L) was added to the mixture after it had been incubated for 20 minutes at 37.5°C. To stop the reaction, 650 μL of a 1 mol/L Na_2_CO_3_ solution was added after the reaction mixture had been incubated for 30 minutes at 37.5°C. A UV spectrometer (UV-2550, Shimadzu, Japan) was used to quantify the amount of released product (p-nitro phenol) at 410 nm in order to determine the enzyme activity.

**4. Anti-Alzheimer's activity**

Using **Ellman's** approach **[37]**, the acetylcholinesterase (AChE) enzyme's inhibition % in this investigation, using donepezil as the reference medication was assessed. Five microliters of 0.5 mM acetylthiocholine (ATCh), five microliters of 0.5 mM 5,5'-dithiobis-2-nitrobenzoic acid (DTNB), and five microliters of each sample (at each concentration) were placed to a 96-well plate with a flat bottom for each run. At 30°C, the mixture was then incubated for ten minutes. The reaction was initiated by adding 5 µL of AChE at a concentration of 0.3 U/mL after incubation, and the absorbance was measured at 412 nm. With the exception of the test sample, a control run involving every component was also conducted. A curve was plotted using a range of sample concentrations versus the percentage of AChE inhibition in order to determine the median inhibitory concentration (IC_50_) of each examined sample.

**5. Anti-arthritic activity**

Using diclofenac sodium as the standard non-steroidal anti-inflammatory medication, as synthesized by **Meera *et al.*** **[38]**, this experiment measured the inhibition percentage (%) of protein denaturation **[39]** and the activity of proteinase enzyme **[40]**. The test control solution, which was made by mixing 0.45 mL of bovine serum albumin (BSA) (5% w/v aqueous solution) with 0.05 mL of distilled water, was mixed with 0.5 mL of protein denaturation inhibitor to determine the percentage. After that, 0.45 mL of distilled water was mixed with 0.05 mL of each sample (at each concentration) to create the product control (0.5 mL). All of the produced solutions' pH values were brought down to 6.3 using HCl (1.0 N). The temperature was raised to 57°C and held there for three minutes after all of the samples had been incubated at 37°C for twenty minutes. Following chilling, the obtained solutions were mixed with 2.5 mL of phosphate buffer. A UV-Visible spectrophotometer was used to measure the absorbance at 416 nm. Calculating the percentage of protein denaturation inhibition is possible. A reaction mixture of 0.06 mg trypsin dissolved in 1 mL of 20 mM Tris HCl buffer (pH 7.4) was combined with 1 mL of each sample (at each concentration) to determine the proteinase enzyme's inhibition percentage. After five minutes of incubation at 37°C, 1 mL of casein (0.8% w/v) was added to the mixture. Two milliliters of 70% perchloric acid were added to stop the process after another twenty minutes of incubation. After centrifuging the hazy suspension, the absorbance of the supernatant was measured at 210 nm using buffer as the blank. A curve was plotted using a range of sample concentrations versus the percentage of proteinase inhibition in order to determine the IC_50_ of each tested sample.

**6. Anti-inflammatory activity**

The suppression of the human and ovine cyclooxygenase-1 (COX-1) and cyclooxygenase-2 (COX-2) isoenzymes, as well as the human recombinant 5-LOX enzyme, was used to assess the *in-vitro* anti-inflammatory properties. Using the COX-1 and COX-2 kit (Cayman, No.: 560131), the inhibition percentages of COX-1 and COX-2 were determined **[41]**. After adding each sample at each concentration to a solution containing 10 μL of COX-1 or COX-2 and 0.1 M HCl buffer, the combination was allowed to sit at room temperature for ten minutes. Next came the addition of Ellman's reagent, 50 μL of HCl, and 10 μL of arachidonic acid. The IC_50_ was calculated using linear regression, and the absorbance was measured at UV-410 nm in conjunction with a blank. The inhibition percentages of 5-Lipoxygenase (5-LOX) were assessed using the 5-LOX kit (No. 437996, Sigma-Aldrich) as proposed by **Huang *et al*.** **[42]**. 90 μL of 5-LOX and 100 μL of de chromogen were mixed with each concentration of the investigated samples, and then 10 μL of arachidonic acid was added. After shaking the mixture for ten minutes, the absorbance at UV-490 nm was measured and compared to a blank. Linear regression was utilized to ascertain the IC_50_.

**Supplementary Table 1.** The individual triplicate data, that were used to calculate statistical correlations among the different *in vitro* biological activities of bioactive glass nanoparticles at a concentration of 1000 µg/mL.

|  | | **TAC** | **IRP** | **DPPH** | **IRP** | **α-amylase** | **α-glucosidase** | **AChE** | **Protein Denaturation** | **Proteinase** | **COX-1** | **COX-2** | **5-LOX** |
| --- | --- | --- | --- | --- | --- | --- | --- | --- | --- | --- | --- | --- | --- |
| **G0P** | 1 | 52.89 | 45.17 | 37.58 | 59.78 | 27.65 | 12.48 | 68.14 | 52.43 | 49.93 | 41.45 | 47.66 | 31.38 |
|  | 1 | 52.71 | 45.05 | 37.39 | 60.03 | 27.71 | 12.52 | 68.32 | 52.58 | 50.08 | 41.58 | 47.82 | 31.21 |
|  | 1 | 52.80 | 45.36 | 37.49 | 59.87 | 27.68 | 12.44 | 67.96 | 52.52 | 50.02 | 41.48 | 47.70 | 31.45 |
| **G1P** | 2 | 60.82 | 51.95 | 43.21 | 68.75 | 28.07 | 12.66 | 62.64 | 60.29 | 57.79 | 42.65 | 49.05 | 32.30 |
|  | 2 | 60.61 | 51.81 | 43.00 | 69.03 | 28.13 | 12.71 | 62.82 | 60.47 | 57.97 | 42.79 | 49.21 | 32.12 |
|  | 2 | 60.72 | 52.16 | 43.11 | 68.85 | 28.10 | 12.62 | 62.46 | 60.40 | 57.90 | 42.69 | 49.09 | 32.37 |
| **G2P** | 3 | 61.74 | 52.73 | 43.86 | 69.78 | 27.68 | 12.49 | 58.14 | 61.20 | 58.70 | 49.05 | 56.40 | 37.14 |
|  | 3 | 61.52 | 52.58 | 43.65 | 70.06 | 27.74 | 12.53 | 58.32 | 61.38 | 58.88 | 49.21 | 56.59 | 36.94 |
|  | 3 | 61.63 | 52.94 | 43.75 | 69.89 | 27.71 | 12.45 | 57.96 | 61.31 | 58.81 | 49.09 | 56.45 | 37.22 |
| **G4P** | 4 | 77.17 | 65.91 | 54.83 | 87.22 | 27.93 | 12.60 | 63.64 | 76.50 | 74.00 | 76.12 | 87.54 | 57.64 |
|  | 4 | 76.90 | 65.73 | 54.56 | 87.58 | 27.99 | 12.64 | 63.82 | 76.72 | 74.22 | 76.37 | 87.83 | 57.33 |
|  | 4 | 77.03 | 66.18 | 54.69 | 87.36 | 27.96 | 12.56 | 63.46 | 76.63 | 74.13 | 76.19 | 87.61 | 57.77 |
